# Novel Neuromodulation Techniques to Assess Interhemispheric Communication in Neural Injury and Neurodegenerative Diseases

**DOI:** 10.3389/fncir.2017.00015

**Published:** 2017-03-09

**Authors:** Samuel S. Shin, Galit Pelled

**Affiliations:** ^1^F.M. Kirby Research Center for Functional Brain Imaging, Kennedy Krieger InstituteBaltimore, MD, USA; ^2^Department of Radiology, Johns Hopkins University School of MedicineBaltimore, MD, USA

**Keywords:** neuromodulation, noninvasive, transcranial magnetic stimulation, transcranial direct current, optogenetic

## Abstract

Interhemispheric interaction has a major role in various neurobehavioral functions. Its disruption is a major contributor to the pathological changes in the setting of brain injury such as traumatic brain injury, peripheral nerve injury, and stroke, as well as neurodegenerative diseases. Because interhemispheric interaction has a crucial role in functional consequence in these neuropathological states, a review of noninvasive and state-of-the-art molecular based neuromodulation methods that focus on or have the potential to elucidate interhemispheric interaction have been performed. This yielded approximately 170 relevant articles on human subjects or animal models. There has been a recent surge of reports on noninvasive methods such as transcranial magnetic stimulation and transcranial direct current stimulation. Since these are noninvasive techniques with little to no side effects, their widespread use in clinical studies can be easily justified. The overview of novel neuromodulation methods and how they can be applied to study the role of interhemispheric communication in neural injury and neurodegenerative disease is provided. Additionally, the potential of each method in therapeutic use as well as investigating the pathophysiology of interhemispheric interaction in neurodegenerative diseases and brain injury is discussed. New technologies such as transcranial magnetic stimulation or transcranial direct current stimulation could have a great impact in understanding interhemispheric pathophysiology associated with acquired injury and neurodegenerative diseases, as well as designing improved rehabilitation therapies. Also, advances in molecular based neuromodulation techniques such as optogenetics and other chemical, thermal, and magnetic based methods provide new capabilities to stimulate or inhibit a specific brain location and a specific neuronal population.

## Background

Although, some high order information processing such as attention and language function are lateralized to a region in one hemisphere, correlation of activity between homotopic regions of the two hemispheres is important for sensory and motor processing (Stark et al., 2008). Magnetic imaging research in neurological diseases has progressed in various areas: detecting specific molecular target (Wadghiri et al., 2013), microstructural injury (Shin et al., 2014), as well as alterations in functional correlation of different regions of the brain (Boly et al., 2009; Iraji et al., 2015). Specifically, in functional imaging the significance of interhemispheric crosstalk has been described in the literature over the years. Imaging researchers have described this as interhemispheric functional connectivity, and it is commonly found in homologous regions of the two hemispheres (Salvador et al., 2005; Margulies et al., 2007). This interhemispheric connectivity is found widely throughout various brain regions: in a functional magnetic resonance imaging (MRI) study of brain regions during resting state, a significant correlation of activities of many bilateral symmetric regions were found (Margulies et al., 2007).

Notable disruptions in interhemispheric connectivity were shown by studies on partial or complete split brain patients. Functional MRI studies show that interhemispheric connectivity between sensory areas is topographically organized (Fabri et al., 2011). Bilateral cortical activation is evoked by communication via different parts of the corpus callosum: gustatory stimuli induces bilateral gustatory cortex activation via anterior corpus callosum, tactile stimuli induces bilateral primary somatosensory area activation via posterior body of corpus callosum, and visual stimulation induces bilateral primary visual cortex activation via splenium. Among patients with partial injury in any region of the corpus callosum, bilateral cortical activation occurred with sensory stimuli only when appropriate region of the corpus callosum was not affected: e.g., bilateral visual cortex activation was preserved only when injury did not affect the splenium (Polonara et al., 2015).

Other task based studies also showed interesting insights into interhemispheric information transfer in callosotomy patients. In a patient that had inadvertent sparing of rostral and splenial corpus callosum there was severe limitation in information transfer: there was no transfer of color, shape and size information, but word information was possible between the two hemispheres (Funnell et al., 2000). However, Kingstone et al. showed that in a patient with complete callosotomy, there was no transfer of abstracted word information between the two hemispheres (Kingstone and Gazzaniga, 1995).

However, a study on resting state connectivity of the two hemispheres in a patient with complete split syndrome showed surprising preservation of interhemispheric interaction (Uddin et al., 2008). This patient had complete forebrain commissurotomy which involved section of anterior commissure, corpus callosum, hippocampal commissure, and massa intemedia for treatment of intractable epilepsy in the past. The authors compared functional connectivity of lingual gyrus, cingulate gyrus, and medial frontal gyrus between the patient and normal subjects, showing that 2 of 3 (lingual gyrus and cingulate gyrus) had normal range of interhemispheric interaction. The authors of this study postulated the reason for preservation of large degree of bilateral activation despite complete commissurotomy as subcortical coordination of bihemispheric activity. Thus in summary, there are topographical functional losses and severe disruption of interhemispheric information transfer in split brain patients, but there is a preservation of limited connectivity between the two hemispheres likely via subcortical pathways.

The functional as well as structural interhemispheric connectivity is extensive and has a major role in modulation of bilateral visual cortices (Nakamura et al., 2008; Schmidt et al., 2010), somatosensory and motor cortices (Killackey et al., 1983; Perez et al., 2007; Baek et al., 2016), and auditory cortices (Imig and Reale, 1980). The initial studies of interhemispheric connections shown in tracing experiments using animal brain preparations are now complemented by structural and functional imaging studies of humans in the recent two decades.

In various pathological states of the brain, whether acute injury or neurodegenerative disease, there are disruptions of interhemispheric connectivity. The strong correlation of interhemispheric connectivity was absent in a minimally conscious patient who had a brainstem lesion (Salvador et al., 2005). Also, subjects with various disease states such as Parkinson's disease (Spagnolo et al., 2013; Hohlefeld et al., 2014; Luo et al., 2015), epilepsy (Thordstein and Constantinescu, 2012; Liu et al., 2013; Lin et al., 2014; Yadollahpour et al., 2014; VanHaerents et al., 2015), stroke (Buchkremer-Ratzmann et al., 1996; Liepert et al., 2000; Takatsuru et al., 2009; Lim et al., 2014; Liu et al., 2015), and peripheral nerve injury (Pelled et al., 2007b, 2009; Pawela et al., 2010; Li et al., 2011; Han et al., 2013) showed alteration of interhemispheric interaction. Understanding the process of the disruption of interhemispheric interaction in brain injury and neurodegenerative disease may give important clues in optimizing rehabilitation and recovery from disease states.

In the past two decades, the neuroscience technology has been rapidly progressing in various forms of neuromodulation research given the potential clinical impact in various neurological diseases. Although most interfaces involved in neuromodulation requires invasive implantation, noninvasive neuromodulation techniques have been developed and tested in both animal and human subjects recently. Given the invasiveness of surgical procedures such as deep brain stimulation (DBS) currently used in clinical subjects, the future possibility of noninvasive neuromodulation technique is an attractive solution. Noninvasive neuromodulation methods such as transcranial magnetic stimulation (TMS) and transcranial direct current stimulation (tDCS) show long-lasting effects on neuronal functions (Gersner et al., 2011; Vestito et al., 2014). Although they have their own inherent problems such as nonspecific effects on the adjacent regions to the target region, technological advancements may increase the region specificity of the noninvasive neuromodulation techniques in the future. Moreover, recent advancements in methods such as optogenetics and chemogenetics have allowed precise modulation of individual neural circuits and subregions of the brain in animal models.

With ongoing research on these novel neuromodulation techniques by many research groups throughout the world, there is a great future potential for this category of neuromodulation. In this review, we will highlight recent advancements of novel methods of neuromodulation in the investigation of interhemispheric activity in brain injury as well as neurodegenerative diseases. In addition to TMS and tDCS, more invasive methods such as optogenetics, chemogenetics, as well as DBS will also be reviewed for comparison.

## Interhemispheric connectivity in DBS studies

Although DBS has been commonly used in treatment of Parkinson's disease, more recently it has been applied in cases of medically refractory depression and obsessive compulsive disease with some degree of success (Lakhan and Callaway, 2010). Also, an important study in 2007 showed that DBS can be used to improve level of function in a severe TBI patient (Schiff et al., 2007). In this study, a TBI patient who was in minimally conscious state for 6 years after the initial injury was subjected to bilateral central thalamic stimulation. Although, the fact that thalamus has widespread projection of axons to various cortical and subcortical structures the mechanism of thalamic stimulation leading to increased neural function in minimally conscious TBI patient was unclear. A preclinical research by Lin et al. (2015) showed that interhemispheric connectivity between the two thalami and dorsal striatum may be increased by central thalamic stimulation. Recordings of local field potentials (LFPs) were made from central thalami, ventral striatum, and dorsal striatum during DBS of central thalami in awake rats in this study. By assessing the synchronous neural activity of these regions by a parameter termed coherence, connectivity of these regions was assessed. These recordings showed that there is increased connectivity between bilateral central thalami, ventral striatum, and dorsal striatum induced by central thalamic DBS. There is also increased intrahemispheric connectivity in central thalamus, ventral striatum and dorsal striatum measured by coherence. Thus, central thalamic DBS enhances both inter and intrahemispheric connectivity. Central thalamic DBS may potentially have similar effects on connectivity of TBI humans and animal models, but this has not been described in the literature yet.

Among Parkinson's disease (PD) patients, scalp electroencephalograms show that higher interhemispheric coherence was associated with increased motor impairment, and DBS of subthalamic nucleus (STN) reduced this interhemispheric coherence at beta band (10–30 Hz oscillations) especially over sensorimotor regions (Silberstein et al., 2005; Weiss et al., 2015). Another report showed that DBS of one STN can induce a significant increase in multiunit spiking activity of a contralateral STN among PD patients (Novak et al., 2009). Despite these studies reporting functional connectivity between the two STNs by several measures, there is no direct evidence of anatomical connection between the two STNs. One potential means of communication between the two STNs can be due to indirect connections: STN and other structures of the basal ganglia are connected to the thalamocortical projections, which then converge via corpus callosum (Hohlefeld et al., 2014). Similarly, interhemispheric striatal connections can be via cortical projection to contralateral striatum (Fisher et al., 1986; Alloway et al., 2009) or substantia nigra projection to contralateral striatum (Douglas et al., 1987; Morgan and Huston, 1990).

### Noninvasive brain stimulation

Animal studies have demonstrated that brain stimulation entailing neurosurgical procedure can improve motor function after brain injury by increasing functional connectivity (Guggenmos et al., 2014) and expansion of cortical representation areas (Plautz et al., 2016). Since brain stimulation can enhance motor function during recovery from stroke in animal models, there have already been feasibility studies of neurosurgical procedures placing cortical or epidural electrodes to improve motor recovery after stroke in humans (Brown et al., 2006; Levy et al., 2008). However, due to the risk involved with surgical complication and cost, more recent interest has been on noninvasive methods that can provide stimulus. Two commonly used methods in recent studies have been TMS and tDCS, which are noninvasive, well-tolerated, and have demonstrated little to no side effects in animal studies as well as clinical trials.

### Transcranial magnetic stimulation

Transcranial magnetic stimulation is a noninvasive method of stimulating specific brain regions. Its application involves placing a coil over the region of interest, and by electromagnetic induction currents are generated in the brain. Since noninvasive stimulation of the brain reduces the risks encountered in surgical patients such as hemorrhage, infection, and the cost of the procedure, TMS has recently gained interest for use in functional and behavioral research as well as rehabilitation research after brain injury (Celnik et al., 2009; Lu et al., 2015) and neurodegenerative disease (Ferbert et al., 1992; Spagnolo et al., 2013; Bocci et al., 2016). It has been used widely in humans (Noh et al., 2012; Wang et al., 2012; Sung et al., 2013; Bocci et al., 2016), primates (Tischler et al., 2012; Mueller et al., 2014), as well as rodents (Barnes et al., 2014; Andreou et al., 2016).

Due to its noninvasive advantages, TMS can be easily used to study interhemispheric plasticity in healthy volunteers without risk of complications such as infection or hemorrhage as in surgical procedures. Though the mechanism of TMS is yet to be clarified, our recent studies suggest that rTMS may lead to spike time dependent plasticity (Banerjee et al., 2017), and therefore an additional stimulus would significantly augment the impact of rTMS on brain function. This particular feature is fundamental to develop TMS-based protocol with long lasting effects and it requires thorough investigation. Variations in TMS protocols that are useful for understanding the interaction between the two hemispheres have been utilized to understand interhemispheric interaction in depth. Theta burst stimulation (TBS) is a protocol of TMS using bursts of 3 pulses at 50 Hz, and each burst stimulus is given at pulses of 5 Hz. There are two types of TBS: continuous TBS (cTBS) which is given as a continuous train over 40 s, and intermittent TBS (iTBS) which is given as 2 s train repeated every 10 s. When applied continuously (cTBS) over areas such as left motor cortex, it can induce long term depression and inhibition of activity. However, intermittent TBS (iTBS) can have the opposite effect of long term potentiation or facilitation of activity (Paulus, 2005). The role of cTBS on interhemispheric as well as intrahemispheric connectivity was observed by applying cTBS over left motor cortex and monitoring changes in electroencephalogram (EEG) oscillations of the contralateral hemisphere (Noh et al., 2015). Inhibition using cTBS over left motor cortex reduced functional connectivity of distant areas of motor network: both interhemispheric and intrahemispheric connectivity was reduced.

Transcranial magnetic stimulation has also been used to study the interhemispheric connectivity among patients with corpus callosum surgical lesions (Meyer et al., 1995). One measure of interhemispheric connectivity is by monitoring transcallosal inhibition, which is an inhibitory control of one motor cortex to contralateral motor cortex by corpus callosum. In subjects with intact anterior corpus callosum connections, application of TMS over one primary motor cortex induced inhibition of electromyographic response of ipsilateral hand muscles. However, patients with lesions in the anterior part of corpus callosum had reduced inhibition. This signified that the inhibitory effect of TMS in subjects with intact anterior corpus callosum was not due to the direct action of the TMS on ipsilateral hemisphere but rather due to the effect of TMS on transcallosal inhibitory function. Another report by Meyer et al. (1998) endorsing this concept demonstrate how TMS can be utilized to study the interhemispheric relationship of various regions of the brain as well as discovering how certain structures important for interhemispheric communication function.

Similar to these experiments, transcallosal inhibition has been studied using paired pulse stimulation protocol that applies TMS bilaterally. Whereas the previously mentioned experiments by Meyer et al. used TMS only on one side for interhemispheric inhibition of voluntary motor activity, paired pulse stimulation applies TMS on two sides: TMS on one side for interhemispheric inhibition (conditioning stimulus) and a second TMS contralaterally to induce muscle contraction (test stimulus). Both protocols used TMS for interhemispheric inhibition, but the paired pulse stimulation protocol uses a second TMS for direct stimulation of muscle contraction instead of voluntary muscle contraction.

A recent example of this protocol was in a study of interhemispheric interaction for scapulothoracic muscle control (Matthews et al., 2013). In this study a test TMS was delivered to the right motor cortex that corresponds to a trapezius or serratus anterior muscle, leading to the corresponding muscle contraction in the left side. Interhemispheric interaction between the two motor cortices is assessed by a delivery of a conditioning stimulus to the contralateral side which can inhibit muscle contraction in an increasing manner as conditioning stimulus intensity increases (Bologna et al., 2012). Thus, the study is performed by initial delivery of the conditioning stimulus, followed by contralateral delivery of test stimulus with 4–8 ms delay. The study by Matthew et al., demonstrated that there is interhemispheric inhibition between the motor cortices that control upper trapezius muscles but not lower trapezius or serratus anterior. Furthermore, some studies have distinguished the two separate phases of interhemispheric inhibition induced by conditioning stimulus: long latency interhemispheric inhibition (LIHI) and short latency interhemispheric inhibition (SIHI) (Udupa et al., 2010). Whereas SIHI occurs at 10 ms following conditioning stimulus, LIHI occurs at 40 ms following test stimulus. With increasing test motor evoked potential amplitude, there was an increase in SICI but not LIHI.

### Transcranial magnetic stimulation studies in stroke

Transcranial magnetic stimulation has been used to study the interhemispheric interaction in pathological states such as stroke. The paired pulse stimulus protocol has been utilized among stroke patients to study interhemispheric interaction, showing that there is decreased interhemispheric inhibition from the infarcted hemisphere to non-infarcted hemisphere (Bütefisch et al., 2008). In another study looking at further details of the interhemispheric interactions between the infarcted and non-infarcted hemispheres as well as LIHI and SIHI using paired pulse stimuli, there was a significant increase in LIHI from non-stroke to stroke side but no change in SIHI (Kirton et al., 2010).

In addition to studying the underlying neurological function and interhemispheric connectivity, the ability to control TMS to inhibit or facilitate target region of the brain can be used to enhance recovery from disease. There have been several prior studies of stimulating premotor or motor cortex in order to manipulate interhemispheric competition and improve motor function. These studies have shown various levels of motor improvement by modulating interhemispheric balance of motor regions (Meyer et al., 1995; Hanajima et al., 2001; Murase et al., 2004; Baumer et al., 2006; Wang et al., 2012). These studies give great promise for the use of TMS when there is interhemispheric imbalance such as stroke.

Interhemispheric inhibition can be explained by bilateral motor regions having a mutual inhibitory control of each other (Murase et al., 2004; Bütefisch et al., 2008; Grefkes et al., 2008). In order to generate unilateral limb movements, there is increased neural activity of contralateral motor areas as well as inhibition from contralateral motor areas to ipsilateral motor areas of the brain. In neurological injury such as unilateral stroke, this interhemispheric inhibitory balance is disrupted (Duque et al., 2005; Volz et al., 2015). Noninvasive stimulation methods such as TMS or tDCS have been used to modulate this imbalance of inhibition.

In one study, patients receiving 3 months of TMS showed improvement in various motor functions such as hand grip strength, keyboard tapping, and NIH stroke scale (Khedr et al., 2009). Also, cortical excitability of the stroke hemisphere was increased and excitability of contralateral hemisphere was decreased which agreed with the concept of using TMS to reverse stroke induced imbalance in interhemispheric inhibitory function.

Further studies on stroke patients have been performed recently by the same group. When 30 patients with post stroke nonfluent aphasia were subjected to rTMS for 10 days followed by language training, there was a significant improvement in language performance by behavioral tests (Khedr et al., 2014). The rTMS consisted of 1 Hz stimulus over the right unaffected Broca's area and 20 Hz stimulus over the left affected Broca's area, showing that dual hemisphere rTMS to suppress transcallosal inhibition from the right hemisphere while stimulating the left hemisphere may improve language performance. There was no improvement in motor function as assessed by hand grip strength in this study. However, the rTMS protocol was different, consisting of targeting bilateral Broca's area (Khedr et al., 2014) instead of contralateral motor area of first dorsal interosseous muscle (Khedr et al., 2009).

More recently, there have been a few other studies showing improvements in motor function after TMS among stroke patients. Similar to the dual hemispheric TMS concept in a study by Khedr et al. a group of 54 hemiplegic stroke patients received contralesional rTMS at 1 Hz and ipsilesional TBS resulting in improvemdent of multiple measures of motor function (Sung et al., 2013). When inhibitory TMS was applied at the contralateral primary motor cortex followed by facilitatory TBS applied at ipsilesional primary motor cortex, subjects had improved motor function after 4 weeks of sessions (Sung et al., 2013). There were improvements in finger flexor muscle strength, Fugl-Meyer Assessment (FMA), Wolf Motor Function test which is a composite measurement of speed, strength, and quality of upper extremity movement. A similar protocol of inhibitory TMS contralaterally and facilitatory TBS ipsilaterally with a long term follow up of 3 months showed long lasting improvement in motor function (Wang C. P. et al., 2014).

Even when ipsilateral excitatory TMS and contralateral inhibitory TMS are applied separately either of the two TMS methods can improve motor function and have lasting effect for 12 weeks of observation period (Emara et al., 2010). Transcranial magnetic stimulation can also be used in conjunction with rehabilitation treatment. By using low frequency repetitive TMS, stroke patients had significantly improved intracortical facilitation, a measure of intracortical synaptic excitability (Mello et al., 2015). Similarly, patients with stroke had improved motor function when they were given inhibitory TMS at contralateral primary motor cortex (Demirtas-Tatlidede et al., 2015) or premotor cortex and primary motor cortex (Wang C. C. et al., 2014). These pilot studies provide promising outlook for using TMS among stroke patients in the future, but the optimal parameters and timeline of stimulation for motor recovery are yet to be determined.

An important insight that the authors of this review suggest is that many of the current studies have selection bias to subjects that respond most strongly to TMS. For example, inclusion criteria by Sung et al. (2013), was only patients between 3 and 12 months, since patients who are more than 1 year post stroke were susceptible to rTMS conditioning based on pilot study observation. Similarly, Emara et al. (2010) had inclusion criteria as patients who showed higher excitability (lower motor threshold). For practical purposes, inclusion of patients who are best responders to rTMS may be necessary as each clinical study cannot include several thousand to tens of thousands of patients without a serious commitment for time and resources of an institution. However, understanding of the patient population who are not as susceptible is a valuable information for the community of neuroscientists because this would give us mechanistic insights which can further be tested in preclinical studies.

In the studies described in this section, a specific interhemispheric pathway involved by TMS stimulation is not specified and is only assumed to transcallossal (Khedr et al., 2014). This is likely due to the previously well characterized disconnection syndromes in patients in corpus callosum injuries, and wide variety of motor and sensory information that is communicated by corpus callosum. However, interhemispheric connection via basal ganglia is also another likely circuit that is modulated by TMS. Coordination of motor functions can occur by bilateral STN communication as mentioned in the DBS section, as well as interhemispheric nigrostriatal connection projections which will be discussed in the following sections describing TMS use in Parkinson's disease research.

### Transcranial magnetic stimulation studies in traumatic brain injury

Although traumatic brain injury can result in various different structural damages, damages to the long white matter tracks are commonly found. Among them, one of the most vulnerable regions of damage is corpus callosum which has a central role in interhemispheric communication (Ljungqvist et al., 2011; Wu et al., 2013). Clinical symptoms of corpus callosum injury are also demonstrated in TBI patients (Falchook et al., 2015), and functional imaging of TBI patients show reduced interhemispheric functional connectivity in hippocampus and anterior cingulate cortex (Marquez de la Plata et al., 2011), as well as primary motor cortex and superior marginal gyrus (Kasahara et al., 2010). These findings have also been substantiated by EEG study with recordings of visual event-related potential, where corpus callosum integrity was evaluated among pediatric TBI patients (Ellis et al., 2016). A measure of corpus callosum integrity used in this study was interhemispheric transfer time, which refers to time it takes for information to cross between the hemispheres by corpus callosum. The TBI subjects in this study had significantly reduced interhemispheric transfer time and poor neurocognitive functions compared to controls. In another TBI study, pediatric TBI patients were studied and a subgroup of the patients showed reduced interhemispheric transfer time that was also associated with impairment of white matter detected on diffusion weighted imaging (Dennis et al., 2015).

Although the dysfunction of interhemispheric communication following TBI has been characterized by these studies, the role of TMS in promoting the recovery from this pathology has not been investigated. There have also been a few case reports and small case series on the use of TMS for the treatment of depression after TBI (Fitzgerald et al., 2011), neurobehavioral deficits (Louise-Bender Pape et al., 2009), and post concussive syndrome (Koski et al., 2015) which showed varying levels of benefit. Investigations on the mechanisms underlying this behavioral improvement show that TMS improves cortical excitability. Cortical excitability was compromised in human patients (Bagnato et al., 2012; Fecteau et al., 2015) and animal models (Li et al., 2014), and motor evoked potential, a measurement of motor cortex excitability, had increased threshold following TBI (Moosavi et al., 1999; Chistyakov et al., 2001). Capitalizing on these findings, TMS was successful in restoring cortical excitability in a rat model of pediatric TBI (Li et al., 2014). In these rats, TMS improved cortical excitability measured by amplitude and number of neuronal spikes, amplitude of local field potential, and functional MRI response. It also increased immunohistological markers associated with plasticity, and it decreased hyperactivity which is a symptom that is often observed in pediatric TBI patients (Li et al., 2014; Lu et al., 2015).

However, these recent studies on the effects of TMS on TBI subjects do not focus on how it affects interhemispheric communication. Given the commonly found corpus callosum damage and evidence of interhemispheric dysfunction in TBI, there is room for further investigation focusing on alteration of interhemispheric interaction.

### Transcranial magnetic stimulation studies in parkinson's disease

Another possible application of TMS to further understand interhemispheric connectivity in a neurodegeneration is in PD research. The important role of interhemispheric interaction in PD is shown by interhemispheric connection of the dopaminergic circuitry: interhemispheric nigrostriatal projections have been identified in experiments where rats were injected with tracer dyes into caudate nucleus with subsequent staining of bilateral substantia nigra (Pritzel et al., 1983b). Although, most of the projections from substantia nigra go to ipsilateral striatum, about 3% of the projection are made to contralateral striatum as evidenced by tracing studies in rats (Douglas et al., 1987). Thus, they have been termed “interhemispheric nigrostriatal neurons.” Tracer studies have shown that these projections are terminated contralaterally in both caudate and putamen (Morgan and Huston, 1990). Also, as chemical lesion of studies of the striatum support the idea that those projections are not branching to both contralateral and ipsilateral side, but that there are two separate projections to ipsilateral side and contralateral side. In addition to corpus callosum connectivity, crossing to the opposite hemispheres is also found at thalamic commissure, indicating that this may be another major component of interhemispheric connectivity.

Asides from anatomical evidence, the important role of interhemispheric interaction in PD were described in PD models using toxic chemicals such as 6-hydroxydopamine (6-OHDA) which causes degeneration of dopaminergic neurons (Pritzel et al., 1983a; Sullivan et al., 1993; Roedter et al., 2001). Unilateral 6-OHDA lesion caused increase in interhemispheric nigrostriatal neurons (Pritzel et al., 1983a). Moreover, Pelled et al. explored interhemispheric connection in the setting of injury using fMRI of unilaterally 6-OHDA lesioned rats (Pelled et al., 2002, 2007a). In these studies, rats had strong bilateral sensorimotor cortex or habenular complex activation, which may be explained by the following mechanism: unilateral lesion of basal ganglia causes overactivation of both hemispheres via extensive interhemispheric connectivity. In addition, resting state imaging of functional connectivity of 6-OHDA lesioned rats demonstrated bilaterally decreased temporal and spatial variances which were indicative of increased synchronization and activity bilaterally (Pelled et al., 2005).

In light of these prior studies characterizing interhemispheric connectivity in animal models of PD, a noninvasive technique such as TMS may be very useful in animal experiments for PD. Especially given the fact that current non-pharmacological interventions for PD are invasive procedures such as DBS, a noninvasive intervention to treat neurodegenerative disease is an attractive strategy. When 6-OHDA injured rats were treated by 4 weeks of daily TMS, they showed improvements in behavior by increased locomotion and decreased amphetamine-induced rotation (Lee et al., 2013). Additionally, there was improvement in biochemical and histologic markers: increased level of neural growth factors and increased number of tyrosine hydroxylase positive dopaminergic neurons. Similarly, TMS given for the same duration reduced apomorphine induced rotatory behavior in rats and also prevented loss of dopaminergic neurons in substantia nigra (Yang et al., 2010).

Prior studies have utilized TMS to study the changes in interhemispheric interaction in PD patients. In PD patients with asymmetric motor symptoms, application of TMS was applied to either hemisphere (Spagnolo et al., 2013). This revealed shorter and smaller ipsilateral silent period, a measure of interhemispheric inhibition in the hemisphere that was more severely affected. Similarly, another experiment utilized paired pulse TMS on opposite hemispheres: a conditioning TMS and test TMS (Ferbert et al., 1992). Test TMS is given to elicit a motor response on the opposite side limb muscle that can be detected by electromyography (EMG). Conditioning TMS is given over the motor cortex contralateral to the side of test TMS, and its role is to inhibit the EMG response induced by the test TMS. By this method using two separate TMS, the degree of interhemispheric inhibition can be directly tested. This has been a useful parameter to study the interhemispheric connectivity in PD patients using TMS. This study showed that subgroups within the population of PD having different motor symptoms had different degrees of interhemispheric inhibition. The PD patients with mirror movements (MM), which are involuntary movements on the contralateral side of the body when one side is moving, had reduced interhemispheric inhibition than PD patients without MM. Thus, there is an association between interhemispheric inhibition and clinical symptoms, and one may hypothesize that dysregulation of interhemispheric inhibition significantly contributes to some of the major symptoms of PD. Although these studies describe interhemispheric connection in the dopaminergic circuitry, in human subjects there are no reports of direct interhemispheric connection such as nigro-striatal projection as it has been shown in rodents.

Based on these findings, application of TMS may be further optimized for use in PD patients for rehabilitation in the near future. Human trials of TMS resulted in a mixed set of results: some have shown evidence of improvement from PD symptoms, but others have shown only limited benefit. Applications of 50 Hz TMS in motor and dorsolateral cortices (Benninger et al., 2012) or 50 Hz TMS in motor cortex only (Benninger et al., 2011) resulted in no change in the measures of movement such as bradykinesia, gait, and Unified Parkinson's Disease Rating Scale. However, there were benefits in mood with motor cortex and dorsolateral cortex stimulation (Benninger et al., 2012). Among PD patients who had apomorphine-induced dyskinesias, rTMS at 1 Hz on supplementary motor area reduced dyskinesias (Koch et al., 2005). The control experiment in this study was performed by the same test subjects undergoing sham 1 Hz rTMS where same intensity stimulus (90% of resting motor threshold) was provided but the TMS coil was titled at 90° off the scalp so that no current was induced in the brain. Sham rTMS group did not have any change in dyskinesia. In another study by the same group, rTMS application to cerebellum reduced levodopa-induced dyskinesias in 20 PD patients (Koch et al., 2009). Control experiment in this study was performed by application of sham rTMS: another 20 patients with stimulus coil tilted 90° away from the scalp. Also, rTMS was applied in the neck of the test subjects 3 months after the original experiment to evaluate for possible confounding effects of stimulating afferent fibers in the neck. In both studies, the experimental group receiving rTMS had significant reductions in dyskinesia compared to control groups.

Although the location of stimulus is a major factor that determines the degree of improvement from PD, differences in duration and parameters of stimuli are also important to consider. A single conclusion is difficult to make from these studies given that multiple methods of functional assessment are used. Moreoever, control experiments for rTMS are also different making direct comparisons difficult. Many of the human studies have used widely different stimulus protocols and further clarification is needed in future studies to find optimal parameters to be applied in PD patients.

### Transcranial magnetic stimulation studies in epilepsy

Antiepileptogenic effects of TMS was demonstrated in animal models (Akamatsu et al., 2001; Rotenberg et al., 2008; Lin et al., 2014; Yadollahpour et al., 2014) and human subjects (Menkes and Gruenthal, 2000; Kinoshita et al., 2005; Thordstein and Constantinescu, 2012; Liu et al., 2013; VanHaerents et al., 2015). Animal models of chemical induced seizure showed that specifically lower frequency stimulation (0.25–2 Hz range) has inhibitory effects on neuronal excitability. This property of low frequency TMS means that it can be used as a tool to provide anticonvulsant effect. Thus, several of these listed case studies and small case series of refractory epilepsy patients showed significant reductions in seizures when low frequency TMS was applied. Specifically, interhemispheric inhibition in epilepsy patients was explored in one study: 18 patients prior to and after temporal epilepsy surgery were assessed for interhemispheric inhibition by applying TMS (Läppchen et al., 2011). There was significant increase in interhemispheric inhibition tested by applying TMS postoperatively, demonstrating how TMS can be used to assess interhemispheric interaction in epilepsy. Since interhemispheric interaction plays a central role in pathophysiology of epilepsy, further studies similar to this can give us more insight into alteration of interhemispheric communication in epilepsy.

The importance of interhemispheric interaction in epilepsy is evidenced by its involvement in seizure propagation. For generalized seizures involving bilateral hemispheres, the important role of corpus callosum in epilepsy is widely accepted (Wieshmann et al., 2015). Surgical resection of corpus callosum has been established as a palliative procedure for these patients given the concept of interhemispheric propagation of seizure activity (Fuiks et al., 1991; Cendes et al., 1993). In addition, the contribution of interhemispheric connections other than corpus callosum such as hippocampal and anterior commissures are also important in interhemispheric communication. Early studies by Bogen et al. in the 60s–70s on patients who received complete corpus callosotomy for epileptic patients showed that anterior and posterior commissures in addition to corpus callosum have important roles in interhemispheric communication. Subjects with anterior or posterior commissurotomies and corpus callosotomies have variety of interhemispheric disconnection syndromes (Bogen et al., 1965).

Prior diffusion tensor imaging results showed white matter derangements in these areas in temporal lobe epilepsy patients with bilateral involvement compared to patients with unilateral disease (Miró et al., 2015). In future studies, further investigation on using TMS to inhibit seizure propagation via interhemispheric connection and exploring pathological interhemispheric communication in epilepsy patients may be explored.

### Transcranial direct current stimulation

Another important method of noninvasive neurostimulation is transcranial direct current stimulation (tDCS) which uses electrodes placed on the surface of the scalp to deliver low amplitude current (1–2 mA) to the subject. Similar to the parameter specific effects of TMS (e.g., cTBS reducing neuronal activity whereas iTBS increasing neuronal activity), the polarity of tDCS can affect the neuronal activity. Whereas cathodal tDCS decreases the excitability of neurons, anodal tDCS increases the excitability. Although the mechanism of tDCS is not thoroughly reported in the literature, recent findings by Monai et al. show that tDCS induces astrocytic calcium surges without affecting local field potential. It also enhances sensory evoked cortical response in an alpha-1 adrenergic receptor dependent manner (Monai et al., 2016). As further research on its mechanism progress, the neuromodulation research community will likely see further enhancements and cell specific targeting strategies in the future.

Transcranial direct current stimulation was used to improve various functional measures in Parkinson's disease (Kaski et al., 2010; Manenti et al., 2014), stroke (Boggio et al., 2007; Di Lazzaro et al., 2014; Meinzer et al., 2016; Valiengo et al., 2016), and traumatic brain injury (Kang et al., 2012; Yoon et al., 2016). This technique has been tested in humans as well as monkey and rats (Baker et al., 1995; Notturno et al., 2014; Lee et al., 2015; Sellers et al., 2015; Yoon et al., 2016). Similar to TMS studies discussed in earlier sections, the alteration in interhemispheric interaction following these disease states can be investigated also by using tDCS. Moreover, interhemispheric connectivity can also be enhanced by application of tDCS. Interhemispheric functional connectivity, as measured by quantitative EEG (qEEG) parameters in healthy volunteers increased following a single session of tDCS (Castillo Saavedra et al., 2014; Morales-Quezada et al., 2014). As a measure of interhemispheric connectivity, these reports analyzed the level of interhemispheric coherence which is defined as a linear relationship between the specific frequency waves (alpha, beta, and theta waves) between the two hemispheres.

In addition, tDCS has been used to induce interhemispheric inhibition similarly to the use of TMS to study interhemispheric inhibition (Läppchen et al., 2011) described in a previous section. Anodal tDCS increased interhemispheric inhibition whereas cathodal tDCS decreased interhemispheric inhibition (Tazoe et al., 2014). This was assessed by the change in the amplitude of motor evoked potentials when either anodal or cathodal tDCS was applied to primary motor cortex. Electromyography measurements of motor evoked potentials were measured in the metacarpophalangeal joints of the test subjects before and after the tDCS. Subjects who received anodal tDCS had facilitation whereas subjects who received cathodal tDCS had inhibition of motor evoked potentials. This demonstrated the potential use of tDCS in analyzing various deficits in interhemispheric interaction for brain injury as well as neurodegenerative diseases in future studies. Another interesting method of tDCS application is via using it in conjunction with TMS: tDCS on one hemisphere and TMS on the contralateral hemisphere can be used simultaneously to increase cortical excitability assessed by motor evoked potential (Park et al., 2014). Future studies will likely focus on finding optimal parameters of tDCS for individual type of disease as well as methods of combining multiple neuromodulation techniques as described here.

Despite these reports of beneficial effects of tDCS, there have been various issues of replicability in tDCS studies. Common variations in skull size or even slight errors and changes in electrode placement can result in significant difference in electric field provided by the tDCS (Minhas et al., 2012; Kessler et al., 2013; Woods et al., 2015). Using a finite element model to compare adult vs. a 12 year old brain, there was a significant difference in electric field (adolescent brain had up to twice the peak electrical field compared to adult brain) attributed to size difference (Minhas et al., 2012; Kessler et al., 2013). Also, another study varied the position of electrodes to study the effects of electrode drift at increments of 5% (1–1.5 cm movement; Woods et al., 2015). This drift commonly occurs since the elastic straps used to hold the electrodes can move unless caution is taken. There is a growing concern of replicability in tDCS studies given such variability, and future tDCS studies need improved methods to standardize and maintain electrode placements to prevent this confounding factor.

## Novel noninvasive neuromodulation techniques

Major advances in molecular and synthetic biology have revolutionized the ability to control cell excitability in living organisms and greatly impacted basic sciences. Tools such as optogenetics and chemogenetics have the advantages of cell type specificity and superior spatial and temporal resolution compared to prior neuromodulation methods. Also, whereas TMS and tDCS affect wide regions of the brain, these novel tools can specifically control a cell type and circuit (Table 1).

**Table 1:** **Novel noninvasive neuromodulation techniques**.

**Technique**	**Subjects**	**Pathology**	**Stimulus characteristics**	**Advantages**	**Disadvantages**
TMS	Humans, Primates, Rats	TBI, Stroke, Parkinson's disease, Huntington's disease, Epilepsy	>1 Hz TMs or iTBS: increases excitability <1 Hz TMS or cTBS: decreases excitability	Fully noninvasive	Effects large brain regions, Potentially difficult to predict area of current, Limited to surface areas, Not cell specific
tDCS	Humans, Primates, Rats	TBI, Stroke, Parkinson's disease, Epilepsy, Alzheimer's disease	Anodal: Increases excitability Cathodal: Decreases excitability	Fully noninvasive	Effects large brain regions, Potentially difficult to predict area of current, Limited to surface areas, Not cell specific
Optogenetics	Primates, Rats	Stroke, Parkinson's disease, Peripheral nerve injury	Channelrhodopsin: Depolarizes neurons Halorhodopsin: Hyperpolarizes neurons	Cell type specific, Location specific, Temporally specific	Requires intracranial injection for gene delivery, Requires implantation of optic fibers

*General characterization of each major technique are described, as well as the subjects of research and their advantages and disadvantages*.

### Optogenetic manipulations of interhemispheric activity

In the last few years the scientific community has seen a surge of high impact research utilizing optogenetics techniques. Although, the genetically targeted use of light activated neural activity is just over a decade old (Boyden et al., 2005; Li et al., 2005; Deisseroth et al., 2006; Aravanis et al., 2007), it has gained much attention due to the capacity to precisely stimulate or silence neurons at a millisecond time scale. Moreover, it can be targeted to specific neuronal subtypes by cre recombinase drivers and viral vectors (Rothermel et al., 2013; Tsien, 2016). Channelrhodopsin (ChR2), a photosensitive ion channel that depolarizes and leads to activation of neural activity when stimulated, was the only protein used during the early days of optogenetic techniques. Further discovery of light activated chloride pump halorhodopsin which hyperpolarizes when photostimulated lead to the capacity to manipulate the neural activity (Zhang et al., 2007). With this discovery, neuroscientists can modulate neural circuits bidirectionally, by either activating it using ChR2 or silencing it by using halorhodopsin.

Given the high spatial and cell type specific nature of optogenetics, this technique can be used to study much more detailed mapping of interhemispheric connections. Optogenetic stimulation was used to map callosal projections previously (Petreanu et al., 2007). This study demonstrated the connection of different cortical layers via corpus callosum projection using a mouse brain slice, by photostimulating ChR2 positive axons on the layer of somatosensory cortex and detecting synaptic currents with whole-cell recording at the contralateral side. In addition, optogenetic techniques can be used for direct modulation of altered interhemispheric connections to aid in research of rehabilitation following injury.

Despite the advantages of cell specific targeting and isolated stimulation of specific neural circuitry, optogenetic techniques still have disadvantages that still warrants consideration. The initial intracranial injection of ChR2 gene and implantation of the optic probe is an invasive process. Optogenetics has been widely applied in preclinical settings. But in order to translate this technology to clinical setting research regarding the safety of gene delivery and miniaturizing the hardware necessary for light activation is required.

Interhemispheric connections play a significant role in post-injury cortical plasticity (Pelled et al., 2007b). In a rat model of peripheral nerve injury, optogenetic approaches were used to manipulate interhemispheric communication and facilitate plasticity (Li et al., 2011). In this study, rats first underwent unilateral denervation of a forepaw, leading to sensory deprivation on the contralateral hemisphere. Rats were the injected with viral agent targeting excitatory pyramidal neurons with halorhodopsin on the healthy somatosensory cortex. Light activation of halorhodopsin in the healthy cortex combined with forepaw stimulation lead to increase of excitatory neuronal activity in the deprived somatosensory cortex. These changes were due to modulation of transcallosal interaction between the two hemispheres. This is a promising concept that can be used to enhance neural recovery from injury where interhemispheric interaction has a major role, such as stroke, traumatic brain injury, and even neurodegenerative diseases.

In the setting of stroke, optogenetic stimulation has also been used to study the cortical connectivity and activity of peri-infarct sites (Lim et al., 2014). The symmetric network present in sham animals was disrupted after stroke, and optogenetic stimulation of the infarcted cortex did not lead to significant depolarizations. In addition, there have been a few recent reports on the concept of using this technique for recovery from stroke (Cheng et al., 2014a,b, 2016). In transgenic mice expressing ChR2, middle cerebral artery occlusion was performed (Cheng et al., 2014b). Rats underwent implantation of optical fiber over the motor cortex affected by stroke and underwent stimulation for 10 days. Following optogenetic stimulation, multiple measures suggested improved recovery: improved cerebral blood flow, increased neurotrophin expression, and better performance on rotating beam test. Similar to the study by Li et al. (2011), these results indicate that the concept of manipulating input to the neurons during reorganization phase following injury can be impactful in the rehabilitation arena. Specifically, interhemispheric communication can be targeted using this very spatially and temporally specific modulation technique during the reorganization period.

Although it has not been reported, recording electrodes were implanted in motor cortex contralateral to the side of stroke, as well as ipsilateral somatosensory cortex and striatum in the study by Cheng et al. (2014b). A characterization of neuronal activity detected by recording electrodes from day 5 to 14 when stimulation took place may provide an interesting insight. Although TMS or tDCS studies to characterize interhemispheric interaction have been reviewed in prior sections, there are only a few current reports on optogenetic studies describing interhemispheric interaction (Table 2). Time dependent and region dependent response to optogenetic stimulation for recovery of interhemispheric communication can be explored in these preclinical studies, as there is less limitation on testing various protocols unlike clinical studies. With optogenetic studies well under way at many research centers, using this technique in various models of acquired injury and neurodegenerative disease will give insight into optimal stimulation strategies for recovery.

**Table 2:** **Preclinical studies using optogenetic methods for interhemispheric communication**.

**Animals (study)**	**Interhemispheric circuit**	**Target area of optogenetic stimulation**	**Outcome parameter**	**Result**	**Future potential research for therapy**
Rats Li et al., 2011	Callosal	Sensory cortex	Cerebral blood flow (optical imaging, fMRI), Local field potential/single unit response (Electrophysiology)	Optogenetic modulation reduced transcallosal inhibition of the contralateral sensory cortex	Peripheral injury research
Rats Fox et al., 2016	VTA/SN - striatal	VTA/SN	Dopamine release (Fast scanning cyclic voltammetry)	Activation of VTA/SN caused contralateral striatal dopamine release	Parkinson's disease research
Mice Sato et al., 2016	Callosal	Binocular zone of visual cortex	Local field potential (Electrophysiology)	Activation of callosal projections caused contralateral visual cortex hyperpolarization in a visual stimuli dependent manner (high contrast visual stimuli caused greater hyperpolarization)	Visual cortex plasticity research
Mice Rock and Apicella, 2015	Callosal	Callosal projections in auditory cortex	Slice action potentials (Electrophysiology)	Activation of callosal projections suppress corticocortical pyramidal neuron activity but facilitated corticocollicular pyramidal neurons	Auditory cortex plasticity research, Auditory rehabilitation

*Other preclinical methods such as thermogenetic and chemogenetic methods have not been reported in the setting of interhemispheric communication. VTA, ventral tegmental area; SN, substantia nigra*.

## Future directions

While optogenetics has a major impact on basic science today, the main drawback is that light does not penetrate the bone. Therefore, optic fibers to deliver the light must be implanted in the target organ (i.e., brain), producing side effects similar to implantable electronic devices. To overcome the need to implant electrodes and optic fibers, other technologies based on synthetic drug delivery (designer receptors exclusively activated by designer drugs (DREADDs); Coward et al., 1998; Zemelman et al., 2003; García-Sanz et al., 2007; Alexander et al., 2009; Magnus et al., 2011), chemical and optical hybrids (Berglund et al., 2013; Land et al., 2014), and magnetic field heating (Huang et al., 2010; Stanley et al., 2012; Chen et al., 2015), are being developed to allow cellular- and region- specific control of cellular function. However, these technologies require the administration of drugs (DREADDs and chemical and optical hybrids) and tissue heating (magnetic field heating). Therefore, the temporal specificity of these techniques is on the orders of minutes to hours, and the inevitable side effects generated from tissue heating remain to be determined. Current efforts are aimed at developing magnetic-field sensitive proteins that can facilitate neuronal function (Qin et al., 2016; Wheeler et al., 2016). Thus, for basic science research, the molecular based neuromodulation technologies will have a long-term impact on studying and controlling neural activity.

When applied to animal models, these technologies could facilitate greater understanding of the role of interhemispheric pathways in disease pathophysiology. Moreover, the ability to modulate each region of the brain in a spatial and cell type specific manner could enable scientists and clinicians to reverse various dysfunctions in intrahemispheric and interhemispheric communications. Additionally, the less-invasive nature of the molecular-based neuromodulation techniques, and the specificity they provide, make them attractive candidates for use in future clinical settings. Although many years of refinement of these methods and further studies on the pathophysiology of these disease entities are needed prior to consideration for clinical use, these techniques are promising tools to be used for neuromodulation in the future.

## Author contributions

Both SS and GP conceptualized the manuscript, drafted the manuscript, revised and finalized it. SS and GP agree to be accountable for all aspects of the work in ensuring that questions related to the accuracy or integrity of any part of the work are appropriately investigated and resolved.

### Conflict of interest statement

The authors declare that the research was conducted in the absence of any commercial or financial relationships that could be construed as a potential conflict of interest.
